# Perceived teacher support, peer relationship, and university students’ mental health: The mediation of reality and Internet altruistic behaviors

**DOI:** 10.3389/fpsyg.2022.999524

**Published:** 2022-12-29

**Authors:** Linlin Feng, Lelin Zhang

**Affiliations:** School of Marxism, Shandong University of Technology, Zibo, China

**Keywords:** perceived teacher support, peer relationship, reality altruistic behavior, Internet altruistic behavior, mental health, university students

## Abstract

Studying in universities is a crucial development stage for students, whose thoughts, feelings, and actions are affected by interactions with their teachers and peers. This study explored the relationships between perceived teacher support and mental health as well as those between peer relationship and mental health among university students, and examined the mediating effects of reality and Internet altruistic behaviors on these relationships. Perceived teacher support questionnaire, peer relationship satisfaction questionnaire, self-reported altruism questionnaire, Internet altruistic behavior questionnaire, and general health questionnaire were administered to 553 university students. Results demonstrated that perceived teacher support and peer relationship positively predicted reality and Internet altruistic behaviors and positively predicted mental health. Reality and Internet altruistic behaviors positively predicted mental health and exerted significant mediating effects on the correlations between perceived teacher support and mental health as well as those between peer relationship and mental health. The male and female students differed insignificantly in the mediating effects of reality and Internet altruistic behaviors. Therefore, no matter for males or females, teachers should provide sufficient support for the students and establish favorable relationships with them. Friendly relationships, comfort, and active communication among peer students are also essential for creating a healthy and harmonious interaction environment. Those various factors of the school have impacts on the mental health of university students through their altruistic behaviors. This study suggests that further emphasis on teacher support and peer relationship is needed to promote the positive development of altruistic behaviors among university students, and ultimately provide a viable contribution to the university students’ mental health interventions.

## Introduction

People’s expectations for the services that universities should provide are increasing, and the society’s requirements for the skills and abilities that university graduates need are increasing day by day, which is not only a challenge to universities, but also a challenge to university students (Chan, 2016). High-quality university environments provide students with excellent educational resources, including professional faculty and harmonious interpersonal relationships. Good interpersonal relationships established by university students can play a supportive and positive role in psychological development (Cassidy, 2004). What’s more, Mallinckrodt and Leong (1992) discussed the question about establishing good teacher–student relationships in higher school can reduce the risk of depression when students feel stressed. In China, the mental health of university students has received notable attention from the government and society (Tang et al., 2021). Similarly, due to the continuous occurrence of university students’ psychological campus tragedies, the international community has also paid great attention to the related problems of university students’ mental health in recent years (Castillo and Schwartz, 2013). Therefore, it is time to look at the impact of campus-level factors on the mental health of university students, which will not only help to improve the educational environment, but also will have important practical implications for mental health interventions of university students.

According to the ecological systems theory, during personal development, individuals gradually become independent from their family microsystems, which are replaced by schools as one of the most crucial environments affecting their development (Bronfenbrenner and Evans, 2000). In relation to the university campus environment, Zhou et al. (2016) have developed the “field-interaction” theory, which identifies the systemic causal relationships that influence the development of university students. As individuals enter the university stage, various fields of material significance enter the student’s life as they are influenced by the university field, and as other fields interact. Teachers and peers, as the most crucial and ultimate factors in the university field, are the nearest others besides the students themselves, and have a lasting influence on the students, especially on their values, cognitive styles, and ways of acting. The various domains have positive or negative resilient influences on university students, who, as educated parties, are somewhat passive in the influence. Therefore, by improving the nearest other in the university field environment, this study gives positive influence to university students, helps or guides their behaviors, and improves their mental health, so that university students can change from a passive and influenced party to a positive and active subject of behaviors, and become the provider and exporter of altruistic behaviors.

The NSSE report^1^ provides us with a new perspective to pay attention to the higher education environment. As described in this report, higher education is undergoing changes, prompting our research to focus on factors such as higher education environment, teacher–student relationships, and student–student relationships. Hagenauer and Volet (2014) concluded that student-faculty relationship plays an important role in higher education because it can influence students’ satisfaction with the course, students’ attitudes and quality of learning, and students’ dropout rates. Social support makes a person feel cared for, respected and loved, and believe that it is part of a system of mutual obligations (Cobb, 1976; Brandon et al., 2010). Social support comes from different sources, such as parental support, teacher support, and peer support, and research has found that social support helps university students adjust successfully to university life (Tao et al., 2000). It can be found that other people who have important impacts on university students, in addition to university students themselves, are teachers and peers who have positive or negative impacts on university students in school. Therefore, our research focuses on the faculty and peer factors as well as the mechanisms (i.e., altruistic behaviors) affecting the mental health of university students.

Teacher’s support refers to the support, response, and help of teachers to students (Wang, 2009). That is also an essential indicator reflecting the quality of the teacher–student interaction and the formation of favorable interpersonal ecologies (Zhang et al., 2020). The main effect model in social support theory indicates that increasing social support can improve the mental health of individuals (Cohen and Wills, 1985; He et al., 2021). Scholars have asserted that students’ perceptions of teacher support considerably affect their self-esteem, self-efficacy, and subjective well-being (Zhang et al., 2019; Sun et al., 2021). Ryan and Powelson (1991) have emphasized it is important to establish a close supportive relationship with adults in the school environment to encourage the internalization of behavioral norms. In the growth of students, teachers can guide them to interact properly with peers, regulate their emotions and take positive steps to deal with things. Students are also provided with a sense of security to explore their surroundings (Marcus and Sanders-Reio, 2001). Thus, students’ perceived teacher support affects their mental health, and teacher support promotes the formation of a harmonious campus environment. Nowadays, universities are required to strengthen the development of scientific research. However, the further differentiation of academic roles of university teachers has prompted teachers to pay more attention to scientific research, which may aggravate the contradictions and conflicts between teachers and students originally formed by hierarchical asymmetry, which may interfere with the socialization process of university students (Zhou et al., 2016), and thus affect the training direction of higher education. The emergence of this social phenomenon reminds us that we should pay attention to the relationship between teachers and students, specifically, the support of university teachers for university students.

In the process that university students gradually become independent of their parents and begin to seek support from their peers, the influence of peers in school becomes stronger (Roach, 2018; Li et al., 2020). The individual-environment interaction model posits that peer interactions form a social environment (Lerner, 2006), where reciprocity among individuals yields greater benefits than those gained from the parent–child relationship (Gorrese, 2016). Favorable peer relationship is conducive to the formation of a favorable peer environment. Mackin et al. (2017) discovered that peer support is a crucial protective factor against stress induced by interpersonal relationship. Active peer relationship reduces behavioral problems in individuals and improves their mental health and satisfaction with life (Huang et al., 2020). When individuals are in an unfavorable peer relationship and experience physical and relational attacks or even campus bullying, they develop a series of negative emotions (Smith et al., 2014; Ji et al., 2020). Kingery et al. (2010) observed that peer refusal and victimization are significantly correlated with anxiety. The group socialization theory based on genetics and sociology proposed that during personal development, individuals consider themselves to be part of a specific peer group (Harris, 1995). They comply with the attitude and norms of the group to ensure similarity with others and therefore easily assimilate to the characteristics of the group (Ma, 2020). University students’ living environment is quite special, and peer relationship is the core of interpersonal relationships. A good interpersonal relationship can help university students maintain a good psychological state. Overall, teacher support and peer relationship exert strong effects on individuals’ mental health. However, few studies have examined how university students’ perceived teacher support and peer relationship are correlated with their mental health, and the underlying mechanisms governing these effects merit comprehensive exploration.

Altruistic behavior refers to the act of generating benefits for others at a cost to oneself (Fehr and Fischbacher, 2003). Many scholars have verified that such behavior improves mental health. For example, Post (2005) proposed the proactive emotion model, and asserted that the proactive emotions induced by altruistic behavior can negate passive emotions and therefore improve mental health. Feng and Guo (2017) discovered that both self-reported altruistic behaviors and peer-reported altruistic behaviors are conducive to the realization of self-value and the improvement of well-being. Post (2014) concluded that a strong correlation exists between the well-being, happiness, health, and longevity of people who are emotionally kind and compassionate in their charitable helping activities. In school environments, the teacher–student relationship is an essential social relationship of students, and is a predictor of altruistic behavior among students. According to the theory of emotional attachment, the positive attachment relationship can promote the emergence and maintenance of individuals’ altruistic behavior to a great extent. Luckner and Pianta (2011) reported a strong correlation between the quality of teacher–student interaction and altruistic behavior among students. A favorable teacher–student relationship and peer relationship are conducive to active interaction among students, increase students’ sense of belonging to schools, and promote an active attitude toward daily life. Moreover, the previous study found that prosocial behavior was related significantly and positively to peer acceptance and perceived support (Wentzel and Mcnamara, 1999). Sullivan (1953) suggested that there are reasons that adolescents are provided with many positive opportunities to learn prosocial skills when in contact with peers, and this conclusion can be supported by extensive literature linking prosocial behavior to peer acceptance in middle childhood (Asher and Coie, 1990). Thus, the harmonious teacher–student relationship and peer relationship can promote altruistic behavior among students (Hughes, 2011; Longobardi et al., 2016). By contrast, when teachers demonstrate unjust behavior and students feel deceived, the students are less likely to demonstrate altruistic behavior (Jiang et al., 2019). Accordingly, the following hypothesis (H1) is proposed: perceived teacher support and peer relationship indirectly predict the mental health of university students through reality altruistic behavior.

The development of the Internet has increased the attention toward Internet altruistic behavior. The large-scale spread of COVID-19 epidemic infectious diseases has affected the well-being and interests of all mankind. Education in various countries has been transformed into online teaching through the Internet. For example, online lectures, online academic life, and necessary social exchanges within the school are offered (Aristovnik et al., 2020). The Internet facilitates a novel teaching approach through online education platforms, and reduces the psychological distance between students and teachers irrespective of the physical distance between them. The teaching approach provides a new model for expanding the teacher–student relationship. Katz (2010) deeply studied the distance learning preference of university students in the early research. When individuals interact with others online, they can also acquire information and gain emotional support from others (Zheng et al., 2021). Teachers can use instant messaging applications or online courses as media to teach students remotely or provide them explanations or emotional support. When individuals receive more social support from the online environment, they experience more active emotions, which are conducive to the generation of altruistic behavior. Therefore, the Internet altruistic behavior not only has an important impact on the positive psychological quality of university students, but also helps to form a social atmosphere of “civilized netizens” (Jiang et al., 2017). Scholars have indicated that reality and Internet altruistic behaviors do not differ in essence, and Internet altruistic behavior can be reality altruistic behavior conducted in an online environment (Zheng, 2013). However, Li and Yang (2018) disagreed with this viewpoint, and Sargeant et al. (2007) also proposed that the prerequisite for conducting Internet altruistic behavior is lower than that for conducting altruistic behavior in other settings. Therefore, individuals are more likely to demonstrate Internet altruistic behavior without forethought (Bennett, 2009). With the development of the Internet, people’s lives are filled with all kinds of Internet information. In the real life, the subject can define his/her own behavior, but in the network life, the individual projects his/her real self into the virtual media world. Bai (2022) pointed out that through narcissistic expression, silent expression, and other ways, individuals present a mirror self that combines performance, openness, and narcissism. In the Internet era, the real self and the performance self constantly play games in the online and offline world. In order to avoid the mirrored self of the subjects, this study makes a detailed distinction between the Internet altruistic behavior and the reality altruistic behavior. Regarding the Internet altruistic behavior of university students, the following hypothesis (H2) is proposed: perceived teacher support and peer relationship indirectly predict the mental health of university students through Internet altruistic behavior.

The present study explored how reality and Internet altruistic behaviors mediate the relationship between perceived teacher support and mental health as well as that between peer relationship and mental health among university students. In the previous investigation, it is found that the level of social support received by boys is significantly lower than that of girls, and girls are more likely to receive material and spiritual support. Interestingly, the mental health level of female university students has not been improved (Xue et al., 2010). The issue of gender differences has always been a topic of concern in the field of education. Buchmann et al. (2008) studied the gender inequality in the field of education in reviews, specifically the gender inequality in the educational performance and achievements of young people from early childhood to adulthood, and they pointed out in the study that women have the best advantage in completing degrees. This shows that education in various countries is affected by gender differences to varying degrees. In the future, with the popularization and development of higher education, the direction of gender differences should be paid more attention to. Therefore, this study believes that it is necessary for us to analyze the gender differences.

## Materials and methods

### Participants and procedure

In this cross-sectional study by means of an online questionnaire survey, convenience cluster sampling was adopted to recruit 600 students from two higher education institutions in Shandong Province, China. A total of 553 samples were retrieved after invalid samples were excluded because there were much missing data on key study variables; thus, the valid response rate was 92.17%. Of the valid samples, 209 and 344 were male and female students, respectively. The participants were aged 18–25 years [mean ± standard deviation (*SD*): 21.00 ± 1.56 years].

The Coronavirus disease COVID-19 is regarded as the worst-hit pandemic of the century to date (Shanafelt et al., 2020). Due to the COVID-19 pandemic, this study distributed and collected online questionnaires through the network platform. University students were recruited by posting advertisements, forum recruitment, and social media publicity. We established group chats of participants to facilitate timely communication with them. The function of adding friends to each other was turned off to prohibit individually communication to ensure the authenticity of the self-report.

The study was carried out as follows: We communicated with the university students on the Internet, and they were completely anonymous during the formal survey. After obtaining their informed consent, we sent questionnaires online and they answered the online questionnaires, and we did not send questionnaires to those who were not willing to cooperate with the investigation. The measures were administrated to the participants by trained research assistants online. The trained research assistants clearly informed the participants before the survey that there is no right or wrong answer to the questionnaire, that there will be no privacy issue related to the participants, and that the information the participants fill in the questionnaire is completely confidential. The participants were also informed that the measures included questions on their beliefs and experiences in daily life and were encouraged to respond to all the items accurately. At the same time, if the participants feel any discomfort during the research process, they can withdraw at any time. The data collection procedures lasted approximately 30 min. The participants completed measures including the demographic questionnaire, the perceived teacher support questionnaire, the peer relationship satisfaction questionnaire, the self-reported altruism questionnaire, the Internet altruistic behavior questionnaire, and the general health questionnaire (total of 105 items). All survey materials are presented in the local language, and the language of the study was Chinese. Upon study completion, each participant received a bonus (CNY 6 = US $0.93) for compensation.

This study was conducted under the approval of the Institutional Review Board (IRB) at the Shandong University of Technology, and was conducted in accordance with the Declaration of Helsinki. Written consent was obtained prior to the research. All participants were over 18 years old, and there were no minors or children involved. This study caused no harm to participants’ physical and mental health, and the results of this study were maintained confidentially.

## Measures

### Demographic characteristics

The participants provided demographic characteristics information including their gender and age, the number of children in the home, and the home location.

### Perceived teacher support questionnaire

The perceived teacher support questionnaire designed by Ouyang (2005) for university students was employed in this study. Previous studies have shown that the questionnaire is suitable for measuring Chinese university students’ perceived teacher support (Zhang, 2012; Yu, 2019). This questionnaire contains 19 items across three dimensions. The learning support dimension contains nine items (e.g., “My teacher is very strict with me when it comes to learning.”); the emotional support dimension contains six items (e.g., “My teacher shows a very gentle attitude toward me.”); and the capability support dimension contains four items (e.g., “My teacher often appoints me to oversee various tasks in the classroom.”). This questionnaire is scored using a 6-point scale, with scores of 1–6 indicating *completely disagree*, *disagree*, *somewhat disagree*, *somewhat agree*, *agree*, and *completely agree*, respectively. The final average scores ranged from 1 to 6. A high score indicates that respondents clearly perceive support from their teachers in learning and daily life activities. In this study, teachers include who are subjectively connected with “me” and all members who have a positive or negative impact on “me” from the perspective of students. They are teachers who have intersection with university students and have an impact or educational significance on university students. In the present study, the internal consistencies of the overall questionnaire, learning support dimension, emotional support dimension, and capability support dimension were 0.95, 0.87, 0.89, and 0.85, respectively.

### Peer relationship satisfaction questionnaire

The peer relationship satisfaction questionnaire designed by Wei (1998) for university students was adopted in this study. Previous studies have shown that the scale is suitable for measuring the peer relationship of university students in the Chinese context, and the reliability is appropriate (Li et al., 2018). This questionnaire contains 20 items. Examples of the items in this questionnaire are “Classmates enjoy spending time with me,” “Classmates are never angry with me,” and “I feel very sad when classmates are ill.” This questionnaire is scored using a 5-point scale, with scores of 1–5 indicating *completely disagree*, *somewhat disagree*, *unsure*, *somewhat agree*, and *completely agree*, respectively. The final average scores ranged from 1 to 5. A high score indicates more favorable peer relationship. In the present study, the internal consistency of this questionnaire was 0.92.

### Self-reported altruism questionnaire

The self-reported altruism questionnaire can be adopted to divide altruistic behaviors into different types (Oda et al., 2013). It contains 21 items across three dimensions: altruism to family members dimension (e.g., “I provide support to my family members when they become ill.”), altruism to friends dimension (e.g., “I tend to listen to the worries and complaints from my friends or acquaintances.”), and altruism to strangers dimension (e.g., “When riding a train, I remind strangers that they can place their luggage on racks.”). Each of these dimensions contains seven items. This questionnaire is scored using a 5-point scale, with scores of 1–5 indicating *completely disagree*, *somewhat disagree*, *unsure*, *somewhat agree*, and *completely agree*, respectively. The final average scores ranged from 1 to 5. A higher score indicates that the respondent is more likely to demonstrate altruistic behavior. The questionnaire has been verified to exhibit high reliability and validity in the context of Chinese culture (Feng and Guo, 2017). In the present study, the internal consistencies of the overall questionnaire, altruism to family members dimension, altruism to friends dimension, and altruism to strangers dimension were 0.97, 0.94, 0.93, and 0.92, respectively.

### Internet altruistic behavior questionnaire

The Internet altruistic behavior questionnaire designed by Zheng (2010) for university students was adopted in this study. This questionnaire contains 26 items across four dimensions: the Internet support dimension, which contains nine items (e.g., “I actively reply to others’ comments.”); Internet guiding dimension, which contains six items (e.g., “I guide other Internet users to use the internet.”); Internet sharing dimension, which contains six items (e.g., “I share with others internet my successful learning experience.”); and Internet reminding dimension, which contains five items (e.g., I expose illegal activities Internet to remind others not to contribute to the same crimes.”). This questionnaire is scored using a 4-point scale, with scores of 1–4 indicating *never*, *occasionally*, *sometimes*, and *often*, respectively. The final average scores ranged from 1 to 4. A high score indicates that the respondent frequently demonstrates Internet altruistic behavior. The questionnaire designed has been established to apply to Chinese university students (Zheng, 2013; Zheng and Wang, 2017; Zheng et al., 2021). In the present study, the internal consistencies of the overall questionnaire, Internet support dimension, Internet guiding dimension, Internet sharing dimension, and Internet reminding dimension were 0.97, 0.94, 0.92, 0.91, and 0.90, respectively.

### General health questionnaire

The general health questionnaire designed by Li and Li (2015) evaluated the mental health of university students was adopted in this study. The Chinese version has been established to apply to Chinese university students (Li and Li, 2015). This questionnaire contains 12 items across two dimensions: the positive health dimension (e.g., “Can you concentrate on completing your tasks?”) and negative health dimension (e.g., “Have you experienced insomnia due to worries?”). Each of these dimensions contains six items. This questionnaire is scored using a 4-point scale, in order to conform to the habit of positive thinking, this study will score the reverse scoring questions in the forward direction, that is, the higher the score, the better the mental health of the university students. The final average scores ranged from 1 to 4. In the present study, the internal consistencies of the overall questionnaire, positive health dimension, and negative health dimension were 0.86, 0.77, and 0.79, respectively.

### Statistical analysis

The collected data were processed using SPSS 19.0 and Amos 23.0. Descriptive statistics and correlational analysis in this study were conducted with the SPSS 19.0. The Amos 23.0 was used to conduct path analysis and multiple-group comparison with maximum likelihood estimation to examine the mediating effects of reality and Internet altruistic behaviors. The significance limit was set at *p* < 0.05. Evaluations of structural equation modeling (SEM) models were conventionally based on the following statistics: the comparative fit index (CFI), the Tucker-Lewis index (TLI), and the root-mean-square error of approximation (RMSEA; McDonald and Ho, 2002; Kline, 2005).

Moreover, the Bootstrap method was used to test the mediating effect by PROCESS (Hayes, 2013), wherein repeated random sampling was used to collect *n* bootstrap samples (*n* = 5,000) from the original data to generate and save n mediating effect values to form an approximate sampling distribution, and the mean path coefficient of the mediating effect was calculated. If the 95% confidence interval of these mean path coefficients did not include 0, the mediating effect was significant (Preacher and Hayes, 2008).

## Results

### Correlation analysis on perceived teacher support, peer relationship, reality altruistic behavior, Internet altruistic behavior, and mental health

Table 1 lists the results of the correlation analysis on perceived teacher support, peer relationship, reality altruistic behavior, Internet altruistic behavior, and mental health. Perceived teacher support was significantly and positively correlated with peer relationship, reality altruistic behavior, Internet altruistic behavior, and mental health (*p* < 0.001). Peer relationship was significantly and positively correlated with reality altruistic behavior, Internet altruistic behavior, and mental health (*p* < 0.001). Reality altruistic behavior was significantly and positively correlated with Internet altruistic behavior and mental health (*p* < 0.001). Internet altruistic behavior was significantly and positively correlated with mental health (*p* < 0.001).

**Table 1:** Correlations among the key study variables.

	1	2	3	4	5
1. Perceived teacher’s support	–				
2. Peer relationship	0.64^***^	–			
3. Reality altruistic behavior	0.28^***^	0.34^***^	–		
4. Internet altruistic behavior	0.33^***^	0.36^***^	0.35^***^	–	
5. Mental health	0.33^***^	0.40^***^	0.20^***^	0.20^***^	–
*M*	4.33	3.78	4.07	2.62	2.55
*SD*	0.79	0.51	0.70	0.72	0.41

^***^p < 0.001.

### Mediating effects of reality altruistic behavior on the relationships between perceived teacher support, peer relationship, and mental health among university students

**Figure 1:**
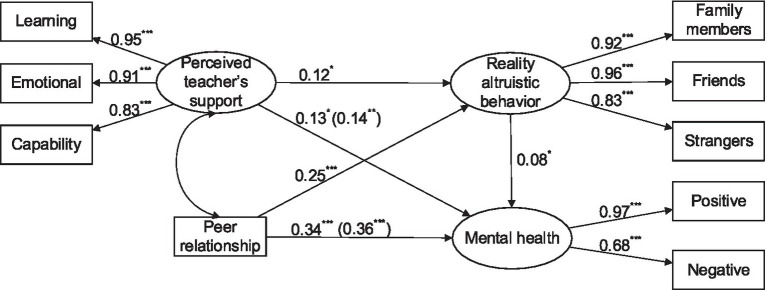
Relationships of perceived teacher’s support, peer relationship, reality altruistic behavior, and mental health. ^*^p < 0.05, ^**^p < 0.01, ^***^p < 0.001.

Amos 23.0 was used to conduct path analysis for testing the mediation roles of altruistic behaviors between perceived teacher support, peer relationship, and mental health in a mediation model (Preacher and Hayes, 2008). Structural equation modeling was first conducted to examine the direct effects of perceived teacher support and peer relationship on mental health. The resulting model exhibited excellent fit [*χ*^2^/df = 1.919, CFI = 0.997, TLI = 0.993, and RMSEA = 0.041]. Perceived teacher support and peer relationship significantly and positively predicted mental health (*β* = 0.14, *p* < 0.01; *β* = 0.36, *p* < 0.001). Next, reality altruistic behavior was conducted as the mediating variable into the direct effect model to create the M_1_ mediating effect model (Figure 1). The M_1_ model exhibited excellent fit (*χ*^2^/df = 2.168, CFI = 0.993, TLI = 0.988, and RMSEA = 0.046). Perceived teacher support directly and positively predicted mental health (*β* = 0.13, *p* < 0.05). It also positively predicted mental health through positively predicting reality altruistic behavior (*β* = 0.12, *p* < 0.05; *β* = 0.08, *p* < 0.05). Peer relationship directly and positively predicted mental health (*β* = 0.34, *p* < 0.001). It also positively predicted mental health through positively predicting Internet altruistic behavior (*β* = 0.25, *p* < 0.001; *β* = 0.08, *p* < 0.05). The proportion of the mediating effect of reality altruistic behavior in total effect was 6.88 and 5.56%, respectively. The overall model explained 22.00% of the variance in university students’ mental health.

A nonparametric percentile bootstrap method was adopted to verify the significance of the mediating effects of reality altruistic behavior (Hayes, 2013). The results revealed that the 95% confidence interval of the mediating effect of reality altruistic behavior on the relationship between perceived teacher support and mental health was [0.1112, 0.1962]. Moreover, the 95% confidence interval of the mediating effect of reality altruistic behavior on the relationship between peer relationship and mental health was [0.2647, 0.3884] (Table 2). The aforementioned results verified that the mediating effects of reality altruistic behavior were significant.

**Table 2:** Bootstrap analysis of the mediating effects.

Independent variable → Mediation variable → Dependent variable	Indirect effect	Boot standard error	95% bootstrap CI	
			Lower limit	Upper limit
Perceived teacher’s support → Reality altruistic behavior → Mental health	0.1537	0.0216	0.1112	0.1962
Peer relationship → Reality altruistic behavior → Mental health	0.3065	0.0334	0.2647	0.3884
Perceived teacher’s support → Internet altruistic behavior → Mental health	0.1527	0.1095	0.1095	0.1959
Peer relationship →Internet altruistic behavior → Mental health	0.3068	0.0336	0.2407	0.3729

### Mediating effects of Internet altruistic behavior on the relationships between perceived teacher support, peer relationship, and mental health among university students

**Figure 2:**
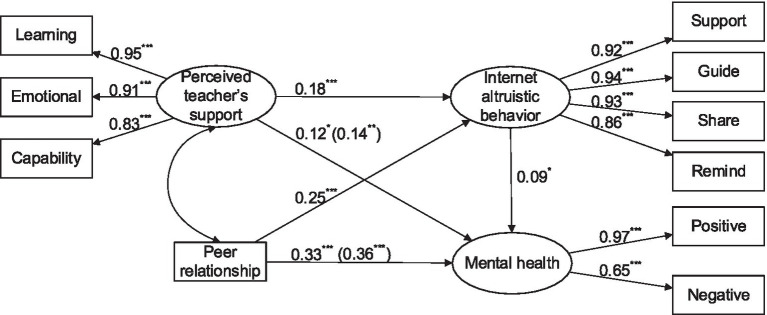
Relationships of perceived teacher’s support, peer relationship, Internet altruistic behavior, and mental health. ^*^p < 0.05, ^**^p < 0.01, ^***^p < 0.001.

Internet altruistic behavior was conducted as the mediating variable into the direct effect model of perceived teacher support, peer relationship, and mental health to create the M_2_ mediating effect model (Figure 2). The M_2_ model exhibited excellent fit (*χ*^2^/df = 2.449, CFI = 0.990, TLI = 0.986, and RMSEA = 0.051). Perceived teacher support directly and positively predicted mental health (*β* = 0.12, *p* < 0.05). It also positively predicted mental health through positively predicting Internet altruistic behavior (*β* = 0.18, *p* < 0.001; *β* = 0.09, *p* < 0.05). Peer relationship directly and positively predicted mental health (*β* = 0.33, *p* < 0.001). It also positively predicted mental health through positively predicting Internet altruistic behavior (*β* = 0.25, *p* < 0.001; *β* = 0.09, *p* < 0.05). The proportion of the mediating effect of Internet altruistic behavior in total effect was 11.89 and 6.38%, respectively. The overall model explained 21.80% of the variance in university students’ mental health.

The nonparametric percentile bootstrap method was used to verify the significance of the mediating effects of Internet altruistic behavior (Hayes, 2013). The results revealed that the 95% confidence interval of the mediating effect of Internet altruistic behavior on the relationship between perceived teacher support and mental health was [0.1095, 0.1959]. Moreover, the 95% confidence interval of the mediating effect of Internet altruistic behavior on the relationship between peer relationship and mental health was [0.2407, 0.3729] (Table 2). The aforementioned results verified that the mediating effects of Internet altruistic behavior were significant.

### Multi-group analysis on gender differences

To explore the moderating effect of student gender in the aforementioned two mediating effect models, multi-group analysis was performed for comparing the differences in the relevant variable relationships between the male and female student groups. Firstly, an unconstrained model was tested (i.e., path coefficients could be estimated freely for the male and female student groups). Next, a constrained model was tested (i.e., path coefficients were identical for the male and female student groups). Finally, the constrained and unconstrained models were compared. The results showed that both models were nonsignificant (Table 3). This finding verified that student gender did not play a moderating role in the M_1_ and M_2_ models. Moreover, it indicated that the effects of reality and Internet altruistic behaviors on the relationship between perceived teacher support and mental health as well as that between peer relationship and mental health differed insignificantly between the two gender groups.

**Table 3:** Goodness-of-fit indices for the multiple group comparison models.

	*χ* ^2^	df	*χ*^2^/df	CFI	TLI	RMSEA	Δdf	Δ*χ*^2^	*p*
Mediating effect model of reality altruistic behavior									
Unconstrained model	85.817	44	1.950	0.988	0.981	0.042	5	2.340	0.800
Constrained model	88.157	49	1.799	0.989	0.984	0.038						
Mediating effect model of Internet altruistic behavior									
Unconstrained model	103.100	60	1.718	0.990	0.986	0.036	5	3.267	0.659
Constrained model	106.367	65	1.636	0.991	0.987	0.034			

## Discussion

When individuals moving from high school to university, in the new campus environment, an important thing they need to face is the relationship between teachers and students as well as between peer students. The previous studies often take primary and secondary school students as subjects, and there were few studies on university students. However, Dong and Yu (2010) found that with the growth of age, students’ academic mood will change accordingly. Therefore, it is necessary to take university students as subjects to verify the relevant contents. This research is relatively novel and innovative, because few studies pay attention to the support of university teachers and most research focus on the previous stages. With the increase of age, changes in many external factors will also lead to many changes in individuals, especially when entering the university, university students are facing the situation of being far away from their families and integrating into the new collective environment. How to let university students gain positive psychological emotions in the university environment needs to be further studied. This article enriches the study of student-teacher relationships and peer relationships from the perspective of the ecological systems theory and the “field-interaction” theory, and proposes new research mechanisms to expand the pathway of improving university students’ psychological well-being, providing theoretical help to focus on the university environment.

In the present study, it was found that perceived teacher support and peer relationship directly and indirectly predicted mental health through altruistic behaviors; these findings are consistent with the hypothesis 1, and the findings supported the study of Zhang (2018). This may be because, for teachers, behaviors such as putting students’ feelings first and actively communicating with them to ensure their study progress are among the ways to express their support. In such cases, students would be more likely to regard their teachers as role models. Bandura (1977) believed that teachers in the school environment are crucial role models who affect the behaviors of students. Therefore, students who perceive higher levels of care and respect from their teachers pay more attention to the conduct of the teachers and view them as role models. The present study concluded that students who are led by positive role models in the school environment are more likely to be less likely to engage in delinquent behaviors and exhibit more altruistic behaviors, which is partly consistent with the findings of Lu et al. (1998). Therefore, teachers investing more support may have greater impacts on students. In the school setting, teachers can increase their own investment in students, use a variety of support tools to make students perceive their teachers’ commitment effectively, extend their influence and prestige, and encourage students to actively engage in altruistic behaviors, which in turn improves their mental health. In addition, for peer relationship, harmonious peer relationships facilitate the socialization process of the individuals. This may be because, in interacting with peers, individuals can learn more about themselves, engage in self-exploration, and gain valid information from others to help them develop. Positive peers can therefore also serve as role models for individuals as they grow. Lu et al. (1998) found that adolescent individuals would show more imitation and performance of altruistic behavior toward role models in teacher–student and peer relationship. At the same time, Cao (2019) suggested that senior pupils may also develop their communication skills and improve their social understanding, thereby improving their psychological well-being.

In addition, this study found that perceived teacher support and peer relationship directly or indirectly predict university students’ mental health through Internet altruistic behavior, thus, hypothesis 2 of this study was also supported. This may be because, on the one hand, the COVID-19 has changed people’s lives and has transformed the main educational environment for university students from reality to online education on the Internet, with university students increasing some of their online behaviors. The role of the teacher is important in the teaching and learning process of online education. Although online learning is personalized and open, enriching the way education is delivered and broadening its audience, prolonged online learning can also lead to deviant behaviors among students. Therefore, increasing teachers’ support for students, organizing a variety of rich online interactive activities, as well as guiding and increasing students’ Internet altruistic behavior in a timely manner can not only increase the practicality and fun of teaching, relieve the anxiety and tension in the context of the epidemic, but also facilitate the transformation of students’ good mindset and promote their mental health. On the other hand, with the development of the Internet, online games have gradually become the main form of entertainment for students. In online games that require collaboration between game players, who know each other in the real world can develop more intimate relationships than do game players who only know each other virtually. Moreover, such online prosocial games considerably improve the peer relationship and prosocial behavior of game players. Prosocial games have their own unique advantages in improving the quality of friendship, that is, in the state of games, social companions are used to enhance mutual benefit. Although scholars have identified negative effects of the Internet (e.g., Internet addiction and cyberbullying; Akin and Iskender, 2011; Olweus, 2012), positive effects of the prosocial games should not be ignored (e.g., Zhang et al., 2016). The favorable teacher–student and peer relationships can serve as drivers of promotion and guidance of online and offline altruistic behaviors to improve the mental health of university students.

Buchmann et al. (2008) found that COVID-19 has a certain gender difference in the impact on men and women. But in this study, no gender difference was observed in the mediating effects of reality and Internet altruistic behaviors, which may be possibly because the school in China emphasized individual development oriented toward collectivism. Student development that emphasizes collectivism places students of different family backgrounds and personal traits in a fair setting, where the students interact and treat each other equally and with respect. Thus, such student development homogenizes the decision-making behaviors of male and female students. For Western individualistic culture, it emphasizes the independence of individual consciousness and logical thinking (Smith et al., 2013), and individuals’ choice and development are unique. Furthermore, the 47th statistical report on China’s Internet development revealed that the numbers of female Internet users and female online game players have increased with time (Jiang, 2021); thus, the difference between the proportions of male and female Internet users is reducing. In addition, Internet users can conceal their gender, which reduces the gender differences in their Internet behavior. Therefore, this study did not observe gender differences in the examined mediating effects.

To sum up, by exploring the students’ teacher support and peer relationship under the COVID-19 pandemic, an effective mechanism (reality and Internet altruistic behaviors) to promote university students’ mental health is proposed. This study found that a campus environment composed of adequate university teacher support and active university peer relationship is conducive to the healthy development of university students. Importantly, teachers and peers can improve university students’ mental health by promoting the implementation of university students’ reality and Internet altruism. The COVID-19 pandemic has changed people’s lives, the relatively closed management of universities needs us to pay more attention to the mental health of university students. Therefore, this study provides strong empirical support for the prevention of mental health of university students under the COVID-19 pandemic.

### Educational implications and contributions

Based on the findings of this study, this paper argues that we should pay attention to the support provided by university teachers to students and the importance of peer relationships, to guide university students to implement altruistic behavior and enhance their mental health. Overall, this paper proposes the following educational guidance: First, in learning, teachers should be responsible for transferring knowledge to students, helping them to solve problems, and acting as mentors to guide them in developing their personalities. In life, teachers should actively get to know their students, give them adequate guidance, and communicate effectively with them to encourage and praise their altruistic behavior in a timely manner to help them grow and develop psychologically. Second, students are guided to develop positive and healthy peer relationships and to exert the influence of positive peer relationships. Students exchange platforms can be established to encourage positive interaction among university students, enable the comprehensive understanding of students’ behaviors, and provide mental health services to university students. In addition to peer relationship, peers’ incentive is a crucial approach for encouraging students. Bao (2020) surveyed 4,461 undergraduate students and conducted in-depth interviews with 18 of these students in China. Some of the students interviewed noted that when they were playing mobile games and they realized that other students were studying diligently, they felt considerable pressure and decided to study hard too. Therefore, we need to value and reflect on the impact of peer relationships, promote healthy competition, build a harmonious school life, and focus on the positive impact of peer relationships. Third, Overton (2013) notes that individuals live in complex and diverse environments interact with each other, and such interactions exert comprehensive and systematic effects on the individuals. Thus, teachers and students should work together to build a harmonious campus environment. The practical significance of this study has two main parts: First, from an individual micro perspective, we focus on improving the mental health of university students, hoping to promote their healthy development through a harmonious campus environment (including perceived teacher support and peer relationship); second, from a macro perspective, this study aims to raise the public’s concern about the campus environment through the study of university students’ mental health, as few researchers have focused on the teacher–student relationship and peer relationship of university students.

### Limitations and future prospects

This study has several limitations. First, a cross-sectional research design was adopted. This type of design is subject to temporal and spatial limitations because it cannot comprehensively reflect dynamic changes in relationships between variables. Accordingly, future studies can adopt longitudinal research designs to examine how dependent variables change with time. Second, because of the COVID-19 pandemic, the data of this study were conducted through the Internet platform. Despite our efforts to create a consistent environment, we could not control all the possible factors that led to a few limitations in the present study. The self-report of participants may be overestimated or underestimated. Therefore, in the future, studies need to improve the research methods to confirm the reliability of the research results. Most of the questionnaires administered in this study were self-reported, therefore, the responses were somewhat subjective, which might affect the interpretation of causal relationships. In the future, we can increase the number of research participants to examine relevant topics from multiple perspectives. Third, this study recruited university students as participants, thus, the study results cannot be generalizable to all social groups, and the conclusions for non-school settings should be drawn cautiously. Future studies can perform social surveys to gain further insights into the characteristics of other demographic groups and thereby obtain more detailed and comprehensive results. This study had more female participants than male participants, which may have affected the results of gender difference analysis. In future studies, researchers should recruit more equal numbers of male and female participants to minimize the effect of gender on the study results.

## Data availability statement

The raw data supporting the conclusions of this article will be made available by the authors, without undue reservation.

## Ethics statement

The study involving human participants were reviewed and approved by the Institutional Review Board (IRB) at the Shandong University of Technology. The patients/participants provided their written informed consent to participate in this study.

## Author contributions

Both authors listed have contributed to data analysis, drafting and revising the article, given final approval of the version to be published, and agreed to be accountable for all aspects of the work.

## Funding

This research was supported by the Humanities and Social Sciences Project of Ministry of Education in China (18YJC190003) and the National Social Science Fund of China (19CSH048).

## Conflict of interest

The authors declare that the research was conducted in the absence of any commercial or financial relationships that could be construed as a potential conflict of interest.

## Publisher’s note

All claims expressed in this article are solely those of the authors and do not necessarily represent those of their affiliated organizations, or those of the publisher, the editors and the reviewers. Any product that may be evaluated in this article, or claim that may be made by its manufacturer, is not guaranteed or endorsed by the publisher.

## Footnotes

^1^ https://nsse.indiana.edu/
